# A novel technique for delineating the effect of variation in the learning rate on the neural correlates of reward prediction errors in model-based fMRI

**DOI:** 10.3389/fpsyg.2023.1211528

**Published:** 2023-12-21

**Authors:** Henry W. Chase

**Affiliations:** Department of Psychiatry, University of Pittsburgh School of Medicine, Pittsburgh, PA, United States

**Keywords:** reinforcement learning, reinforcement sensitivity, prediction errors, fMRI, general linear model

## Abstract

**Introduction:**

Computational models play an increasingly important role in describing variation in neural activation in human neuroimaging experiments, including evaluating individual differences in the context of psychiatric neuroimaging. In particular, reinforcement learning (RL) techniques have been widely adopted to examine neural responses to reward prediction errors and stimulus or action values, and how these might vary as a function of clinical status. However, there is a lack of consensus around the importance of the precision of free parameter estimation for these methods, particularly with regard to the learning rate. In the present study, I introduce a novel technique which may be used within a general linear model (GLM) to model the effect of mis-estimation of the learning rate on reward prediction error (RPE)-related neural responses.

**Methods:**

Simulations employed a simple RL algorithm, which was used to generate hypothetical neural activations that would be expected to be observed in functional magnetic resonance imaging (fMRI) studies of RL. Similar RL models were incorporated within a GLM-based analysis method including derivatives, with individual differences in the resulting GLM-derived beta parameters being evaluated with respect to the free parameters of the RL model or being submitted to other validation analyses.

**Results:**

Initial simulations demonstrated that the conventional approach to fitting RL models to RPE responses is more likely to reflect individual differences in a reinforcement efficacy construct (lambda) rather than learning rate (alpha). The proposed method, adding a derivative regressor to the GLM, provides a second regressor which reflects the learning rate. Validation analyses were performed including examining another comparable method which yielded highly similar results, and a demonstration of sensitivity of the method in presence of fMRI-like noise.

**Conclusion:**

Overall, the findings underscore the importance of the lambda parameter for interpreting individual differences in RPE-coupled neural activity, and validate a novel neural metric of the modulation of such activity by individual differences in the learning rate. The method is expected to find application in understanding aberrant reinforcement learning across different psychiatric patient groups including major depression and substance use disorder.

## 1 Introduction

Many simple mathematical psychological models make predictions for how a perceptual, emotional or cognitive process might vary on a trial to trial basis, in responses to stimuli presented within a given trial, as well as the history of stimuli that the individual has experienced. Examination of the trial by trial variation in neural correlates of these model predictions has had a substantial if not revolutionary effect on human neuroimaging methodology (O'Doherty et al., 2007; Cohen et al., 2017). One reason is an increase in sensitivity to detect coupling of a predicted psychological process with a neural one: it is often the case that neural responses to stimuli are expected to vary in a complex way. Without modeling this variation in terms of the model predictions, neural responses to a given stimulus would be treated as equivalent, or collapsed within arbitrary and inefficient categories. Another reason might be an increase in specificity, as suitable designs might allow different parametric terms to unpick independent processes which might be confounded if simpler categorical approaches are used.

Building on the success of this method, the examination of how the neural correlates of these psychological processes might vary across patient groups has become a key methodological approach within the computational psychiatry field. For example, numerous recent studies have sought to describe the neural correlates of reward prediction errors (RPEs) derived from reinforcement learning (RL) models (Garrison et al., 2013; Chase et al., 2015b; Fouragnan et al., 2018), and investigate individual differences with respect to psychiatric symptomatology. Within this framework, a typical study of this type might seek to describe the dynamics of neural activation within a conditioning paradigm in terms of an RL model, and then compare activations between patients and healthy controls (e.g., Kumar et al., 2008; Murray et al., 2008; Rose et al., 2014; Culbreth et al., 2016; Lawson et al., 2017). Within such studies, the analysis of trial by trial variation in neural activity following the predictions of RL is a central component.

There has been a steady increase in the number of studies applying these techniques, and more recently model-based approaches been applied to tasks which have been widely used in psychiatric research such as the monetary incentive delay task (MID: Cao et al., 2019). In the present work, I will address the construction of the RL model used for trial by trial modeling. Briefly, all RL models have at least one free parameter—the learning rate (alpha)—and there are three main strategies for estimating this parameter which are typically employed for the analysis of functional magnetic resonance imaging (fMRI) data. First, a reasonable estimate of the learning rate (e.g., 0.2) can be selected for all participants (e.g., Kumar et al., 2008; Lawson et al., 2017). Second, a learning rate parameter for each individual can be estimated from each participant’s behavioral data, if such data are available (e.g., Schonberg et al., 2010). A third method might be considered a hybrid of the previous two: a summary learning rate is obtained by fitting behavioral data for each individual subject, and which is then combined into a single estimate of the overall learning rate for the group, and then applied to all participants’ fMRI data (e.g., Cohen, 2007). Other methods which might concurrently fit neural and behavioral data have been developed (Turner et al., 2013) but have not, as yet, been widely adopted in the computational psychiatry field to my knowledge.

**Figure 1:**
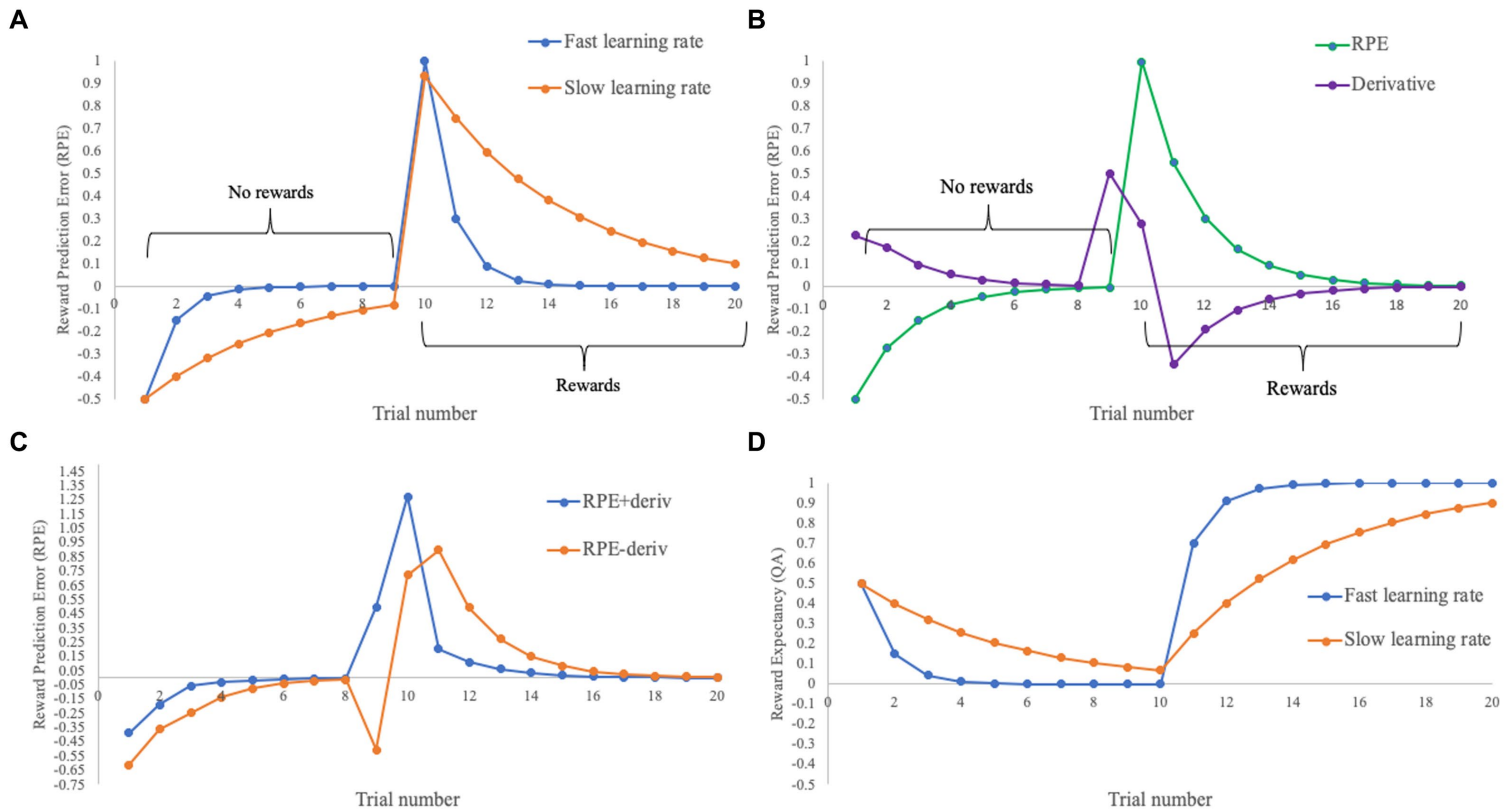
Changes in RPE through trials including non-reward (1–9) and reward (10–20) outcomes for a simple conditioning paradigm. Initial Q set to 0.5, lambda set to 1. **(A)** Variation of RPE with fast (0.7: blue) and slow (0.2: orange) learning rates. Note the slower decline in RPEs in the slow learning rate from trial 10 onwards. **(B)** Variation in RPE with an intermediate learning rate (0.45: green), and its derivative obtained from the *gradient* function in MATLAB (purple). **(C)** The intermediate learning rate RPE displayed in **(B)**, with the derivative added (blue) or subtracted (orange). Note that this broadly captures the predictions of an RPE model with a faster learning rate when added or slower learning rate when subtracted, albeit with some inaccuracies especially on trial 9. **(D)** Variation in the value of the cue (Q) for the fast (0.7: blue) and slow (0.2: orange) learning rates.

At first glance, these three different approaches (fixed, individual, group) would appear to make radically different assumptions about an underlying neural process, which might have a powerful impact on the resulting observations. In addition, the individual method might be expected to be differentially sensitive depending on the precision of the behavioral data available, adding another layer of interpretational complexity. However, a provocative analysis by Wilson and Niv (2015) questioned the extent to which the accuracy of learning rate parameter estimation was actually critical for the modeling of RPE-coupled neural responses. Briefly stated, their argument is that the overall shape of the learning curve, and thus of elicited RPEs (see Figure 1), is generally similar regardless of the magnitude of the learning rate parameter. Moreover, within typical model-based fMRI methodology, there is actually an extra free parameter in addition to the learning rate—the beta parameter from the general linear model (GLM) describing the coupling of the presumed psychological quantity (e.g., RPEs) to neural activity. This free parameter can, in many cases, successfully compensate for inaccuracies in the estimation of the learning rate parameter from behavioral data. Concretely, if the learning rate parameter is under-estimated—set lower than the ground truth value—the RPE-coupled beta parameter will generally be relatively small because the model will predict that larger RPEs will tend to be elicited for longer than they actually are, and vice versa if it is over-estimated. In the original demonstrations of the neural correlates of reward prediction errors (e.g., O'Doherty et al., 2003), the key question of interest was whether RPE-coupled beta parameters would be greater than zero across a group of participants: within Wilson and Niv’s analysis, this type of test would be robust to even substantial misspecification of the learning rate.

However, a more recent study examined the question from an individual-differences perspective—more relevant for computational psychiatry—and here the exact magnitude of the beta parameter is more important (Katahira and Toyama, 2021). Of course, if RPEs are strongly represented in a given brain region in one group but absent in another, conventional methodology may be adequately sensitive to this difference. But any loss of precision will generally act to reduce sensitivity. Of the three methods of learning rate estimation already described, it is hard to know which is preferable overall: the advantage of the fixed model is that any bias should be equivalent across participants, so individual differences might be reflected in the bias. Alternatively, there is potential for additional precision afforded by the individual fitting method, as well as a metric of individual learning rates which can help characterize the dimensions of behavioral and neural difference across individuals more thoroughly (Katahira and Toyama, 2021).

At this stage, it is important to note that the learning curve has two key components: the asymptote, and the rate of rise to asymptote. The two are difficult to differentiate in many of the learning paradigms employed in fMRI, but clearly reflect different psychological constructs. Asymptotic behavioral output is a measurement of the efficacy of the reinforcer, as might be reflected in behavior within a progressive ratio paradigm, for example (Hursh and Silberberg, 2008; Bradshaw and Killeen, 2012), or in preferences between different reinforcer types (Madsen and Ahmed, 2015). Note that even in a preparation with a single response-reward relationship, responding for a reward would be relative to competing behaviors including relaxation, grooming and exploration (Rodgers et al., 2010). More efficacious reinforcers motivate a relatively greater level of responding to obtain the reinforcer, but not in a state-independent way: reinforcement efficacy may be susceptible to manipulations such as deprivation/satiety (Balleine, 1992) or other contextual factors (Stoops et al., 2005). In a classical conditioning paradigm, asymptotic output of other metrics of conditioning including autonomic measures (e.g., skin conductance or heart rate) might also depend on the efficacy of the reinforcer (Neumann and Waters, 2006). Reinforcer efficacy can be defined in within-subject terms (e.g., a preference for one reinforcer over another in a given individual), and also between-subjects terms (e.g., a particular dimension of individual differences enhances or diminishes preference for a given outcome).

By contrast, the learning rate (alpha) determines the rate at which asymptotic output is reached. It has less influence over the asymptote itself (at least with sufficient numbers of trials). Within the Rescorla-Wagner model (Rescorla and Wagner, 1972), reinforcement efficacy would be represented by the lambda parameter, with QA*_t_* representing the current value of the stimulus at time t and outcome being 1 for rewards and zero for no rewards.


(1) $${QA}_{t+1}={QA}_{t}+\left(\mathrm{alpha}\times {RPE}_{t}\right)$$

(2) $${RPE}_{t}=\left(\mathrm{lambda}\times {\mathrm{outcome}}_{t}\right)-{QA}_{t}$$

It is important to note however that lambda is often neglected in computational model fitting—probably for the reason described above—i.e. that it is difficult to distinguish alpha and lambda in typical paradigms employed in fMRI paradigms, and one or other is selected in the interests of parsimony. A further impediment is that lambda and a parameter reflecting the preference for exploration over exploitation (temperature—see Equation 3) have an essentially identical impact in the majority of two-alternative forced choice paradigms (Huys et al., 2013), and are often not identifiable using typical behavioral methods. This does not mean, however, that temperature and lambda are not conceptually distinct constructs, but following from the above definition of lambda in terms of reinforcement efficacy, if behavioral output is dominated by the pursuit of a particular reinforcer, exploratory behavior will be reduced. It might be possible to dissociate lambda from temperature using preferences between distinct reinforcers rather than options which differentially predict the same reinforcer (e.g., Hogarth and Chase, 2012). Temperature is also unlikely to be relevant in a classical conditioning paradigm in which any exploratory behavior is non-existent or incidental.

It may be worth reconsidering lambda in the modeling of RPEs measured by fMRI for two main reasons. First, the distinction between alpha and lambda is germane to questions that are relevant to psychiatry—for example, whether individual differences in trait anhedonia are better understood as an altered acquisition of the incentive value of outcomes but similar asymptotic performance, or a more general suppression of incentive value (Chase, 2021). Second, I have argued that there are in fact two free parameters involved in the modeling of neural coupling to prediction errors, even though it is the learning rate which receives most attention. It may be that these two parameters are suboptimally identified by a typical fMRI study of RPE-correlates of learning, which has led to an emphasis on one or other. More generally, it is possible to imagine scenarios in which there is very rapid learning about a reinforcer which has very limited effects on behavior in terms of behavioral choice or other measures of incentive value (i.e., high alpha, low lambda). Alternatively, if predictive cues are not very discriminable, or have undergone pre-exposure manipulations (e.g., Granger et al., 2021), it is possible to imagine slowed learning about highly efficacious reinforcers (i.e., low alpha, high lambda).

The present work constitutes a reconsideration of the parameter fitting debate relevant for examination of individual differences in neural correlates of RL-derived prediction errors, within the context of a two parameter (lambda/alpha) rather than a one parameter (alpha) model. First, using simulations, I demonstrate that the beta parameters describing RPE/neural coupling produced by a GLM model are better associated with individual differences in lambda than alpha. Second, I show that adding a second regressor, a derivative term, into the GLM has a good specificity for individual differences in alpha. Further tests were conducted to evaluate the approach in the presence of fMRI-like signal and noise, and its psychometric properties (e.g., test–retest reliability).

## 2 Methods

### 2.1 Overview

Briefly the present work involves sets of simulations of two very simple conditioning paradigms, which is assumed to elicit reward prediction errors (RPEs) and corresponding neural activation (Schultz et al., 1997). These simulated data serve as “ground truth” neural activity, and are modeled using a GLM-based strategy which is widely employed in neuroimaging research (Poline and Brett, 2012). Beta parameters derived from this general linear model fit were then tested to determine the extent to which they reflect individual differences in lambda and alpha. This analysis is based on the assumption that participants vary in the magnitude of their lambda and alpha, and that this reflects a meaningful individual difference relevant to psychiatry – a point which is considered further in the discussion.

### 2.2 Paradigm

**Figure 2:**
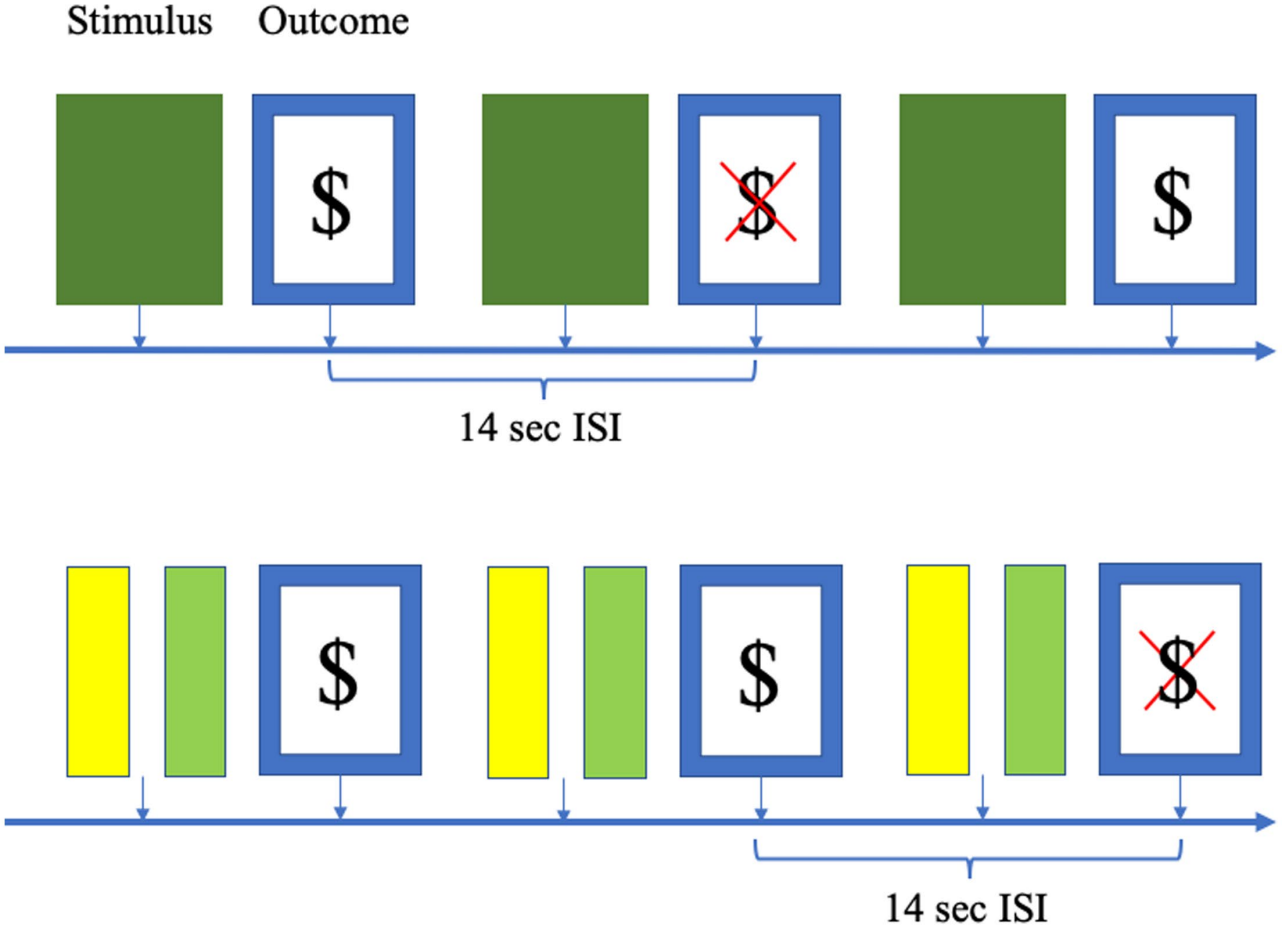
Schematic to show the simulated paradigms employed for the classical conditioning (top) and instrumental paradigms (bottom). In the conditioning paradigm, a cue (here represented as green) predicts an outcome [here represented as a monetary reward ($) or no monetary reward (red X)]. The probability of a rewarding outcome (as opposed to no reward) starts at 0.5, but drifts up or down through the experiment. The instrumental paradigm has a similar design, except that there are two cues which the simulated participant must choose between. These cues have the same cue-outcome probabilities as the conditioning paradigm. Interstimulus interval (ISI), the distance between the outcome events, is 14 s in all cases.

The primary paradigm employed for the simulations was a simple classical conditioning paradigm with a single stimulus which is rewarded with a starting probability of 50% (see Figure 2). The probability of reward on a given trial drifted from trial-to-trial at a rate which is allowed to vary across individuals between 0 and 0.4 (so the probability of reward on trial n + 1 is the probability at trial n plus normally-distributed noise scaled by the drift rate). A second, instrumental paradigm was used for confirmation purposes. This included two stimuli which varied (independently) with the same starting probability of reward (0.5) and drift rate (0–0.4), and participants made a choice between them on each trial.

In typical fMRI experiments, the effect of interest (in this case, RPE), is elicited within a more or less complex paradigm with various components including visual stimulus presentation, anticipation, motor responses, and so on. In this case, I assumed that these components are perfectly modeled out, and only the RPE signal remained. In addition, I assumed that low frequency physiological or scanner-related drifts, which are normally addressed by high pass filtering in fMRI analysis, were perfectly removed from the data. The “ground truth” timeseries was constructed where an RPE response was elicited once every 14 s, with the data being sampled at one data point every 2 s.

### 2.3 Simulations

#### 2.3.1 Standard approach

For the basic simulations, “ground truth” fMRI data was simulated using the classical conditioning paradigm, varying alpha of the Rescorla-Wagner model (Equations 1 and 2) from 0.2–0.7, and lambda from 0.75 to 1.25. The range of alpha was designed to reflect the core predictions of reinforcement learning, i.e., incremental trial and error learning (Sutton and Barto, 2018). If the learning rate is very low (~0.1), the RPE regressor is effectively colinear with reward and no reward outcomes, so the differential predictions of reinforcement learning vs. responses to outcomes are unclear (Rohe et al., 2012). Moreover, if the learning rate is very high (~0.8–0.9), performance on an instrumental design would approximate a win-stay/lose-shift behavior, which can be conceptualized in terms of a working memory process (Frank et al., 2007). The range of lambda was chosen to match that of alpha, to facilitate comparison, centered on 1 as that is the value which is typically used in the literature. It should be noted that the principles arising from the simulations are assumed to generalize outside of this range of parameters, but that the chosen range was thought to be of most theoretical and practical significance for reinforcement learning modeling. The outcome is set to 1 for wins and 0 for non-wins for all simulations. The stimulus value (“QA_t_” in Equation 1) was updated according to Equations 1 and 2, having been initialized for the first trial at 0.5. RPEs were held to represent the activity of midbrain dopamine neurons on a given trial (Schultz et al., 1997). With an inter-stimulus interval (ISI) of 14 s between each outcome and 200 trials (during which activation was set to zero), a timeseries of 1,400 data points was constructed. 5,000 participants’ worth of data were simulated.

These data were modeled at the participant level using another RPE timeseries of the same length. There are two major approaches which are typically used for analyzing such data in the literature: employing a fixed or an individualized learning rate. Lambda was set to 1 in all cases, while alpha was either approximated either by a fixed learning rate, or by obtaining an approximation for each participant (individual). Both approaches were tried: a fixed learning rate of 0.2, or a more (±0.05) or less (±0.1) precisely estimated individualized learning rate. The resulting RPE timeseries, generated using these parameters, was normalized for each participant. Thus, the ground truth fMRI timeseries acted as the dependent measure, and the RPE timeseries derived from the fixed or individualized learning rate was the independent measure within a regression model implemented in MATLAB (the *regstats* function). The resulting beta parameter describing this relationship (i.e., the RPE-coupled beta parameter) was used as a dependent measure within a multiple regression analysis, in which its magnitude was predicted by lambda, alpha and the drift rate across 5,000 simulated participants. 5,000 is much larger than the sample sizes which have typically been used in fMRI studies of individual differences in RPE activations, and this number was chosen to avoid concerns about unstable estimation of effect sizes in small samples (Grady et al., 2021).

A similar strategy was adopted for the instrumental version. Here, two stimuli (A and B) were reinforced with the same contingencies as above (a starting value of 50%, with the same drift rate: 0–0.4). On each trial, the simulated participant would select one of the options, and then receive the outcome, indicating whether they won or lost. A softmax function (Equation 3) was used to determine the probability of selection of one of the two options (in the example, the probability of selecting A at time t), with a parameter controlling the consistency of responding. To avoid confusion with the beta parameters derived from the GLM, this is termed “inverse temperature” for the purposes of the present work, and represented by Θ in Equation 3. This parameter was varied with a flat distribution from 0–5, and acted to multiply the values of A and B.


(3) $$p\left(A_{t}\right)=\frac{e^{\Theta QA_{t}}}{e^{\Theta QA_{t}}+e^{\Theta QB_{t}}}$$

#### 2.3.2 Development of the derivative parameter

Two methods were designed to capture the independent effect of the learning rate on the shape of the RPE regressor. As described in the introduction, an additional regressor representing the rate of change of the RPE regressor across trials was expected to compensate for the effect of mis-estimation of the learning rate on the RPE regressor. In all cases, “ground truth” data were generated using the same parameters as in Section 2.2.1 (e.g., the same range of alpha and lambda).

The first approach, which is used primarily within the simulations, was a derivative term, obtained using the *gradient* function in MATLAB (see Figure 1). Here, a basic RPE regressor was generated with an alpha value of 0.45, which was then used to generate the derivative. Both were included in the GLM following normalization.

For the second approach, the difference between RPE timeseries for a given run generated with a high learning rate (alpha = 0.7) vs. a low learning rate (alpha = 0.2) was calculated, and this served as the derivative regressor. The main RPE regressor was the mean of these two timeseries. As the difference and the main RPE regressors were often substantially correlated using this method, an initial orthogonalization step was performed by regressing out the effect of the latter from the former. Different neuroimaging software has different methods of orthogonalizing regressors (Mumford et al., 2015), but this point is not considered further for these simulations.

In the two cases, as before, the main RPE regressor and the derivative regressor were normalized before inclusion in a multiple regression model as independent measures, and then the beta parameters derived from each were used as dependent measures for two subsequent analyses. First, the main RPE-coupled beta parameter was predicted by alpha, lambda, and drift rate, as before, but now the derivative-coupled beta parameter was also predicted by the same measures in a separate multiple regression model.

The first method—the derivative regressor—was also tested within the instrumental version described above. This analysis followed the same pattern as previously, with drift (0–0.4), alpha (0.2–0.7), lambda (0.75–1.25), and inverse temperature (0–5) all being varied across the 5,000 participants. 200 trials, with an ISI of 14 s, were included per participant as before.

A further analysis was performed with the derivative approach in which the outcome (win/no win) timeseries was also added to the (within-participant) regression model as a third regressor in the GLM (i.e., RPE, derivative, win/no win). Again, the same parameters were used as before.

### 2.4 Generalization to more representative simulated fMRI data

These initial analyses represent only proof-of-concept analyses as the RPE timeseries does not capture any of the characteristics of real fMRI data. First, neural activity as measured by fMRI unfolds over time in a manner corresponding to the hemodynamic response function (HRF). Second, fMRI signal is affected by various sources of noise, including physiological and thermal components. These noise sources can be approximated by pink noise (Bullmore et al., 2001; Akhrif et al., 2018). Thus, to simulate more realistic fMRI data (Equation 4), I convolved the “ground truth” fMRI RPE timeseries with the canonical HRF from SPM software (Friston et al., 1998), and then added pink noise to this timeseries (varying the autocorrelation of the noise (a in 1/f^a^) from 0.8 to 1.2, and the ratio of signal to noise (SNR) from 2 to 4, across simulated participants – SN = 0.66–0.8). This range of SNR was derived from a study of Frassle et al. (2017): I used a smaller range toward the lower end of their SNR range so as not to adopt an overly optimistic estimate of fMRI noise. The same parameters of alpha, lambda, drift rate, and the ISI were used as in previous sections.


(4) $$\begin{array}{l} \mathrm{Simulated}\;\mathrm{fMRI}\;\mathrm{timeseries}=SN\\ \times \;\left(HRF\;\mathrm{convolved}\;RPE\;\mathrm{timeseries}\right)\\ +\;\left(1-SN\right)\times \left(Z\;\mathrm{tranformed}\;\mathrm{noise}\right) \end{array}$$

**Figure 3:**
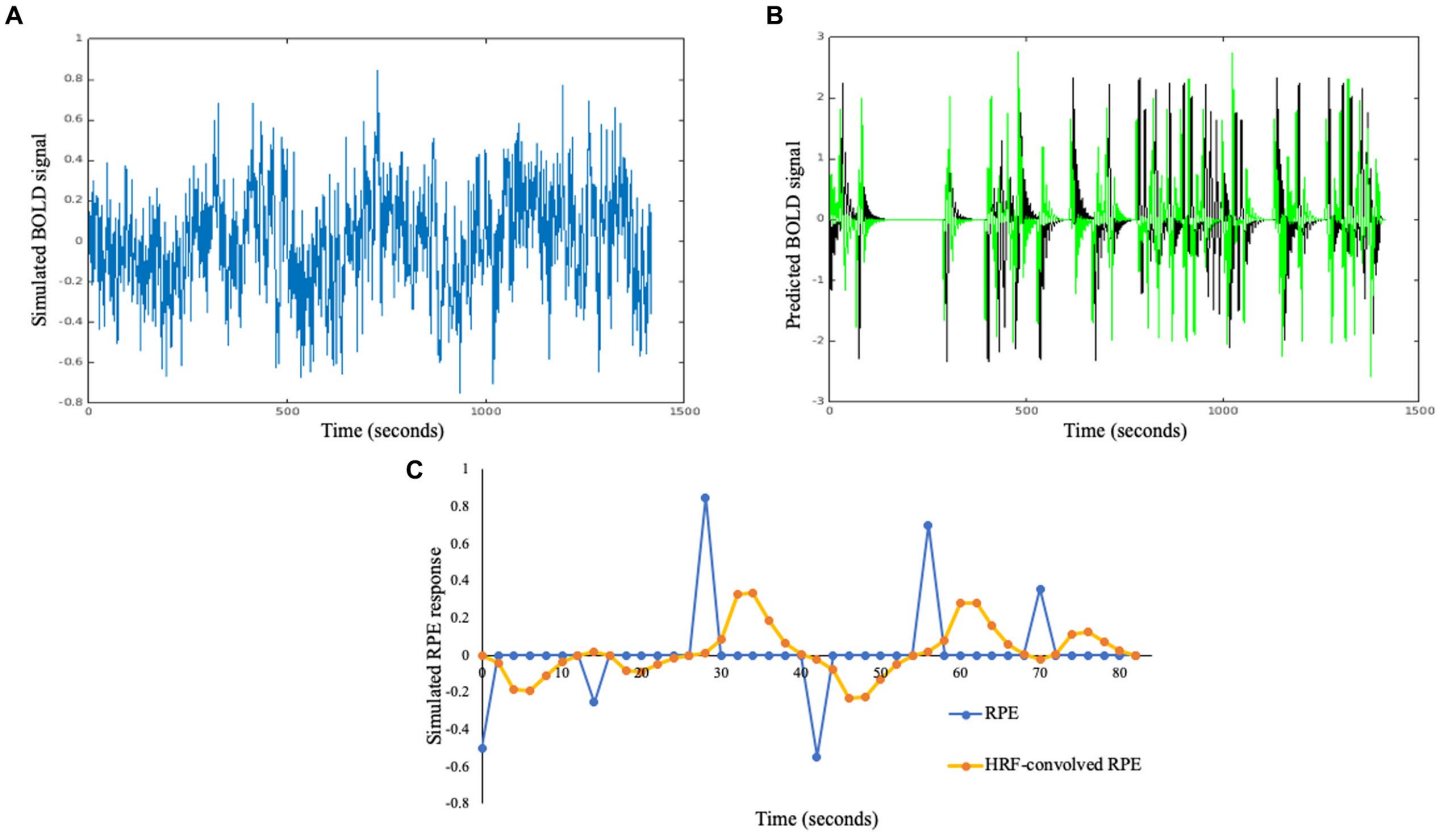
**(A)** Example of a simulated “ground truth” BOLD signal including RPE responses and fMRI-like noise. **(B)** Examples of RPE (black) and derivative (green) regressors that would be used to analyze the ground truth timeseries. **(C)** Figure shows an example of raw RPE signal and HRF-convolved signal for six trials/82 s worth of data for one participant.

This physiological timeseries was modeled using a GLM including RPE and derivative (gradient method) regressors, as before, but also including a linear trend. For these new analyses however, both regressors were convolved with the HRF before normalization (see Figure 3 for example timeseries). In addition, an AR (2) regression model (Monti, 2011) was fit for each subject using the ARIMA function in MATLAB.

The broader purpose of these analyses was to examine whether effect sizes of the type that might have been reported within the literature already might be seen in the presence of realistic fMRI noise. The effect sizes in question were those describing the coupling of lambda/alpha to the RPE/derivative time series. The analysis was run across five different task durations (25/50/100/200/400 trials), with 5,000 simulated participants per cell. Two regression models were constructed in which the RPE or derivative-coupled beta parameters were predicted by alpha, lambda, drift rate, SNR and noise autocorrelation. In addition, Pearson’s correlations of associations between RPE/derivative and alpha/lambda were used to compute effect sizes, and to form the basis of an inferential test to compare the relative magnitude of these associations (Dunn and Clark, 1971) using the *cocor* method (Diedenhofen and Musch, 2015).

Using similar parameters, I evaluated the test–retest reliability of the method in the presence of realistic fMRI noise, generating 2 blocks of 100 trials using identical parameters per participant. Overall, the same ranges of parameters as previously within this section. The same AR (2) model was fit for both blocks, and the intra-class correlation (ICC (3,1)) of RPE-coupled and the derivative-coupled beta parameters for the two blocks was calculated across 5,000 participants. The same analysis was rerun on the same simulated data, but without the derivative regressor.

Two further analyses were run using similar parameters across the 5 trial durations as previously. First, I sought to investigate the extent to which alpha or lambda could be determined from RPE-coupled or derivative-coupled beta parameters. However, for this analysis, I also relaxed the assumption that the HRF scaling was scaled at 1 and identical for all participants: here, I allowed it to vary from 0.5 to 1.5, in addition to the other parameters which were varied as before. Thus, if the HRF scaling parameter was 0.7 for one participant, the HRF would be multiplied by 0.7 before convolution with the RPE timeseries. The impact of this change was assessed using zero-order Pearson’s correlations, and partial correlations in which the HRF scaling parameter was partialled out.

Finally, within this analysis framework, I also sought to determine the extent to which adding the derivative parameter actually improved GLM fits at the subject level. This type of analysis is often not performed in fMRI data (but see Rohe et al., 2012), at least partly because the same GLM usually fit across all brain voxels. A model might therefore be optimized for some voxels but not others. However, here I was able to evaluate whether Bayesian Information Criterion (BIC) was improved for simulated participant’s data by adding the derivative parameter, and what experimental factors were related to any improvement. I predicted that in cases where the true alpha diverged more strongly from the fixed alpha used for the RPE regressor (0.45), a greater advantage of the model including the derivative would be seen. By contrast, when the true alpha was close to the fixed alpha, adding the derivative would have little benefit and would be penalized by BIC. A dichotomous dependent measure was created which represented a binary improve/not improve metric, depending on whether BIC was lower for the RPE&derivative model relative to the RPE model. A logistic regression was then run in which the various variables (lambda, HRF scaling, drift rate, SNR, and noise autocorrelation) manipulated in the simulations were included as independent measures. In addition, rather than alpha being included, a measure of alpha’s distance from 0.45 was included (|alpha-0.45|). Further interaction measures were included between absolute distance of alpha from 0.45 with noise autocorrelation, and with SNR, respectively. For this analysis all independent measures were z transformed before running the model.

## 3 Results

### 3.1 Basic association of lambda vs. alpha with RPE-coupled beta parameter

In the initial simulation, I tested whether individual differences in alpha (0.2–0.7) or lambda (0.75–1.25) would predict the magnitude of the GLM-estimated RPE-to-neural beta parameter in the Pavlovian paradigm, fixing alpha to 0.2 and lambda to 1. Individual differences in lambda [*t*(4996) = 201.39, *p* < 0.001] and to a much less extent, alpha [*t*(4996) = −45.53, *p* < 0.001], but not the drift rate of the paradigm (*t* < 1) predicted the magnitude of the RPE beta parameter. See Supplementary Table S1 for a full overview of all the analyses.

A very similar set of findings were observed in the instrumental version of the paradigm. Using a fixed alpha of 0.2, a strong association between lambda and RPE-coupled beta parameter was seen [*t*(4995) = 174.52, *p* < 0.001], a weaker relationship with alpha [*t*(4995) = −31.041, *p* < 0.001] and absent associations with drift rate and inverse temperature (*t* < 1).

Next, I considered a case where an independent estimate of alpha is available: for example, from behavioral data. Two simulations were performed using the same parameters as previously, but with an accurately estimated alpha (estimated alpha differed from the ground truth alpha with a flat distribution bounded at ±0.05), and or a less accurately estimated alpha (error = ±0.1) for each simulated participant, rather than a fixed alpha. Again, the RPE beta parameter was strongly related to lambda in the high precision [*t*(4996) = 176.066, *p* < 0.001] and low precision [*t*(4996) = 181.023, *p* < 0.001] individualized alpha analyses, whereas the alpha was less strongly related to the RPE beta parameter [high *t*(4996) = 27.27, *p* < 0.001; low *t*(4996) = 31.45, *p* < 0.001], and the drift parameters were not (*t* < 1.45, *p*’s > 0.16).

This initial finding suggests that individual differences in lambda track with the GLM-derived RPE beta to a much greater extent than alpha. Put simply, if an individual has a large lambda, they will show large fluctuations in neural responses to rewards, driven by RPEs. Likewise, an individual with a small lambda would show much smaller variation in neural responses to reward and consequently RPE-coupled beta. With respect to alpha, this has a smaller effect. At the very least, this analysis suggest that lambda deserves further attention as a determinant of GLM-coupled RPE betas, and might be seen as a primary candidate for a psychological construct which underlies variation in neural responses to RPEs.

### 3.2 Addition of an extra derivative parameter in the GLM

For a given lambda, alpha affects the rate at which the asymptote, specified by lambda, is reached. This means that there might only be a few trials for which its effect is clearly observed, and that adding an extra regressor to capture the variance which is not well modeled on these trials might enhance the specificity of the basic RPE regressor for variation in lambda, and generate a new regressor which might reflect variation in alpha more specifically.

Two methods were tested for doing this. The first employed the derivative of the RPE, obtained using the *gradient* function in MATLAB, in addition to the RPE. The same simulation parameters were employed as before, with a fixed alpha of 0.45 to obtain the RPE regressor. In this case, the RPE beta parameter was more specifically related to lambda [*t*(4996) = 120.88, *p* < 0.001], rather than alpha [*t*(4996) = 34.99, *p* < 0.001] or the drift rate (*t* < 1). By contrast, the beta parameter associated with the derivative was more specifically related to alpha [*t*(4996) = 395.44, *p* < 0.001] rather than lambda (*t* < 1.15) or the drift rate (*t* < 1).

To provide evidence of a more specific relationship with RPE as opposed to simple responses to win outcomes, I added a third regressor representing wins or no wins. Again, the RPE beta was related to variation in lambda [*t*(4996) = 155.66, *p* < 0.001], but less with alpha [*t*(4996) = 87.040, *p* < 0.001] and not with drift rate (*t* < 1), while the derivative beta was related to alpha [*t*(4996) = 343.66, *p* < 0.001] but only weakly with lambda [*t*(4996) = 7.34, *p* < 0.001] and not with drift rate (*t* < 1). The win/no win regressor was related to lambda [*t*(4996) = 8.69, *p* < 0.001] and alpha [*t*(4996) = −143.89, *p* < 0.001] but not drift rate [*t*(4996) = −1.85, *p* = 0.064].

I also generalized the method to a simulated instrumental paradigm. A very similar pattern of findings was seen, in which lambda was associated most strongly with the RPE beta parameter [*t*(4995) = 152.29, *p* < 0.001], but more weakly with alpha [*t*(4995) = 30.18, *p* < 0.001], and alpha was associated most strongly with the derivative beta parameter [*t*(4995) = 275.69, *p* < 0.001], while lambda was not strongly related to the derivative beta [*t*(4995) = 2.55, *p* = 0.011]. Neither the drift rate nor inverse temperature were strongly associated with either (*t* < 2.87, *p*’s > 0.004).

I also explored an alternative strategy for achieving the same result—a “high/low” method in which the mean and difference of two RPE time series obtained using a high and a low learning rate parameter, respectively. The beta parameter associated with the basic RPE time series, here represented by the mean, was again correlated with lambda [*t*(4996) = 183.28, *p* < 0.001] but less with alpha [*t*(4996) = 20.98, *p* < 0.001] and not with the drift rate (*t* < 1), while the beta parameter associated with the difference regressor was correlated with alpha [*t*(4996) = 395.88, *p* < 0.001], and less with lambda [*t*(4996) = 6.80, *p* < 0.001] and not with drift rate (*t* < 1).

### 3.3 Generalization of method to realistic fMRI-like signal

The simulations above demonstrate the capacity for basic relationships between simulated RPE regressors and a ground truth generated across two types of conditioning paradigm, but do not demonstrate a potential for generalizations to more realistic neural signals nor across different durations of data collection. For example, they do not include appropriate methods for modeling timeseries autocorrelation (Davey et al., 2013). Finally, they also do not include a formal statistical comparison of the magnitude of different associations (i.e., the association of RPE-coupled beta parameters with alpha vs. lambda). Here, I generalized the ground truth (neural) time series to include fMRI-like noise, and for the ground truth RPE responses to be convolved with the HRF. A model including an RPE regressor and a derivative (both also convolved with the HRF) was fit to this simulated neural time series (see Figure 3 for an overview). The crucial point for these simulations is whether (1) the relationship between lambda and RPE beta parameter, and (2) the relationship between alpha and the derivative beta parameter are representative of the magnitudes of effect sizes that have been observed in individual differences studies. Simulations were performed across different acquisition durations (and thus trial numbers), as, in practice, this can vary markedly across studies.

**Figure 4:**
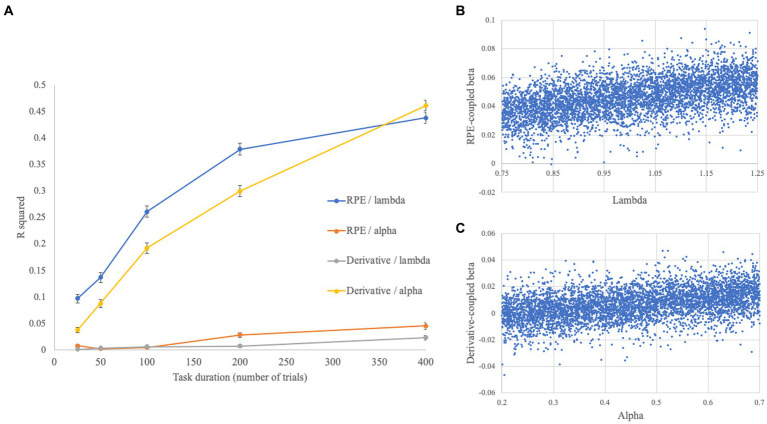
**(A)** Zero-order relationships between lambda/alpha and RPE/derivative-coupled beta parameters, represented in terms of R^2^, across different task durations. Error bars reflect the standard error. **(B)** Figure displays an example of relationship between lambda and RPE-coupled betas, derived from an analysis including 100 trials worth of data in 5,000 simulated participants. **(C)** Figure displays an example of relationship between alpha and derivative-coupled betas from the same analysis as **(B)**.

Findings from these simulations are displayed in Figure 4. Briefly, relationships between lambda and RPE [*t*(4994) = 17.39, *p* < 0.001], and between alpha and the derivative [*t*(4994) = 11.74, *p* < 0.001], were associated with small/medium effect sizes at 25 trials (*d* = 0.48/*d* = 0.33 respectively), which increased into the large range with increasing paradigm duration (*d* = 2.31/*d* = 2.09 at 400 trials). Significant differences were observed, by statistical comparison of correlations, between the strength of these respective associations even at 25 trials (i.e., lambda/RPE > alpha/RPE: *z* = 10.82, *p* < 0.001; alpha/derivative > lambda/derivative: *z* = 8.47, *p* < 0.001), and the statistical magnitude of these differences increased with increasing numbers of trials (up to *z* = 43.12/*z* = 39.63 respectively). Other relationships between the individual differences parameters (drift rate, SNR, and noise autocorrelation) with the RPE and derivative regressors were often non-significant and associated with small effect sizes, although significant associations between SNR/RPE-coupled beta parameter were observed across all trial durations [e.g., *t*(4994) = 6.33–28.54 for 25–400 trials]. Overall, the results of these simulations revealed a dependency on the number of trials in the paradigm, with effect sizes for the key associations of interest (lambda/RPE, alpha/derivative) increasing with increasing numbers of trials per participant.

I also considered whether the derived beta parameters were consistent across two separate “runs” of 100 trials, and thus what the test–retest reliability of the two regressors might be. Relatively low ICCs (i.e., in the “poor” range) were observed for the RPE-coupled [ICC (3,1) = 0.27: CI: 0.25–0.30] and for the derivative-coupled [ICC (3,1) = 0.17: CI: 0.15–0.20] beta parameters, across 5,000 simulated participants. ICCs were also calculated for the RPE-coupled beta parameter from the same data with the derivative parameter not included, with a very similar ICC being observed [ICC (3,1) = 0.27: CI: 0.25–0.30].

Next, I investigated allowing the HRF scaling to vary across individuals. This change affected the relationship between lambda and RPE-coupled betas, with somewhat smaller zero-order relationships being observed across the 5 paradigm durations [r’s increasing from *r* = 0.25 (25 trials) to *r* = 0.42 (400 trials)]. However, the magnitude of these relationships could be recovered by partialling out the HRF scaling parameter [partial r’s increasing from *r* = 0.29 (25 trials) to *r* = 0.74 (400 trials)]. By contrast, the relationship between alpha and the derivative-coupled betas were very similar whether the HRF scaling parameter was partialled out or not: zero-order correlation increased from *r* = 0.21 (25 trials) to *r* = 0.68 (400 trials); partial r’s increased from *r* = 0.21 (25 trials) to *r* = 0.70 (400 trials).

In terms of model comparisons, BIC generally favored the simpler RPE model rather than the RPE&derivative model, with only 11.52% of the 5,000 simulated runs for which BIC favored the latter model at 400 trials (1–2% for 25–100 trials; 4.82% for 200 trials). However, consistent with my predictions, runs on which the RPE&derivative model was preferred were associated with alphas which were more different from 0.45. The basic pattern of findings was generally observed across all trial numbers, but unsurprisingly were much stronger for 400 trials, and these will be reported due to a reduced risk of instability with logistic regression. Absolute alpha difference from 0.45 increased preference for the RPE&derivative model [*t*(4991) = −13.65, *p* < 0.001], as did increasing lambda [*t*(4991) = −6.86, *p* < 0.001], increasing HRF scaling [*t*(4991) = −13.93, *p* < 0.001], increasing SNR [*t*(4991) = −9.14, *p* < 0.001] and noise autocorrelation [*t*(4991) = −4.39, *p* < 0.001]. However, absolute alpha difference from 0.45 did not interact significantly with noise autocorrelation or SNR (*t*’s < 1.2). In summary, the RPE&derivative model was generally not preferred compared to the simpler RPE across the great majority of simulations, consistent with Wilson and Niv (2015). However, it was more often preferred when alpha diverged more sharply from 0.45, and in conditions which favored characterizing RPE signals relative to noise (e.g., more trials, high lambda, high SNR, high HRF scaling).

## 4 Discussion

The question of how best to fit models of psychological processes to neural data is an enduring one. Much of the work into this question in humans has been conducted within reinforcement learning paradigms (O'Doherty et al., 2007; Cohen et al., 2017), and has found areas of application in the study of psychiatric patients. In the present work, I reconsider the question of the mapping of reinforcement learning output parameters, in this case the basic reward prediction error (RPE) signal, on to neural activation as measured using fMRI, and how the precision of estimation of the alpha value can affect this. The overall idea is that participants, for example, clinical participants, might vary in terms of a reinforcement-relevant parameter, which in turn may be reflected in individual differences in RPE-related activation. Model fitting is important in this context insofar as it allows a more precise mapping between an underlying clinically-relevant parameter and RPE-coupled neural activation.

In contrast to the majority of previous work, I considered the lambda parameter in the RW equation, in addition to the alpha parameter. Briefly, lambda controls the reinforcing efficacy of a particular reinforcer. It is often not considered for fMRI studies perhaps because it is not easily identifiable and distinguishable from learning rate or temperature parameters in behavioral paradigms, particularly in 2-alternative forced choice (2AFC) paradigms which are often employed (Daw, 2009; Huys et al., 2013; Chase, 2021). In practice, it controls asymptotic output, e.g., maximum behavioral response rate.

I found that the beta parameter derived from an RPE-regressor derived from a simple RL model with a fixed learning rate correlated much better with variation in lambda than with alpha. This state of affairs was very similar if alpha was estimated for each participant, either at a relatively lower or higher level of precision. Together, these represent the strategies typically adopted within the field for examining RPE-related activation (Cohen, 2007; Chase et al., 2015b). The fact that lambda is a powerful predictor of RPE-related activation implies that previous findings which have observed relationships of such activations with clinical measures, for example, may have done so because the clinical measures are associated with lambda (see Lebreton et al., 2019 for a critical detailed discussion of this point). Certainly, at least, the likelihood that the clinical measure is related to lambda is greater that the likelihood that it is related to alpha—in the absence of any other information.

This finding paved the way for a new hypothesis. In many tasks, although learning rate does play a role in influencing behavior of course, in many paradigms its effect can be diminished with extended training as the participant reaches asymptotic performance. The effect of variation in learning rate is mostly seen then in the first few trials as the participant starts to learn the reward contingencies, or following a change in the stimulus- or response-outcome contingencies. In simple terms, it controls the shape of the learning curve—its rate of change—while lambda controls the asymptote (Rescorla and Wagner, 1972). In a neuroimaging context, this is analogous to the hemodynamic response function (HRF) and the derivative of the HRF: the latter can be used as a supplementary predictor to explain variation in the shape of the HRF across individuals (Friston et al., 1998). The inclusion of both the standard HRF and the derivative affords greater flexibility in modeling variation in the true HRF (Calhoun et al., 2004; Handwerker et al., 2004; Fournier et al., 2014). The overall logic of this strategy is akin to a Taylor series expansion (Friston et al., 1998).

In light of this, I hypothesized that a similar strategy of identifying a derivative of the RPE regressor might provide another regressor which reflects the shape of the learning curve, and show relationships with alpha. After computing a timeseries representing RPE for a given learning rate, I calculated the derivative of that timeseries. Two different methods were tried (derivative/gradient method, high/low difference method), and both gave highly similar findings: namely that individual differences in lambda were strongly associated with the magnitude of the RPE-coupled beta parameter, while individual differences in alpha were associated with the derivative beta parameter. Alpha was also more modestly related to the RPE-coupled beta parameter, but lambda was unrelated to the derivative beta parameter.

I generalized this method to more realistic, synthetic fMRI data, modeling physiological and thermal noise as pink noise, and fitting an AR(2) general linear model to each participant’s data, finding compatible associations of lambda/alpha with RPE/derivatives. This approach was inspired by a previous review (Monti, 2011), and was used here because it is, to my knowledge, *not* implemented in any of the more popular GLM-based models used for fMRI data analysis (Chen et al., 2013; Corbin et al., 2018; Olszowy et al., 2019). Whether the derivative method works as expected within any fMRI analysis software is beyond the scope of the present work. What the findings do show is that the method can model meaningful variation of simulated physiological RPEs in the presence of fMRI-like noise, using a GLM model which can capture the autocorrelational properties of such noise.

Crucially, effect sizes observed within these simulations are quite similar to those which would be expected for positive results in the literature. Although effect size estimation is difficult within fMRI, it is probably sensible to assume *a priori* that most effects identified by fMRI should be considered medium sized (Poldrack et al., 2017). Thus, much of the extant literature on reward function across different clinical groups (Radua et al., 2015; Luijten et al., 2017; Chase et al., 2018; Keren et al., 2018; Janouschek et al., 2021) would then be somewhat underpowered given the sample sizes which are typically used (often in the range of 15–30 participants per group). However, the findings show that variation in lambda exerts a *large* effect on RPE-related activation given largely realistic assumptions (an assumption regarding HRF scaling was also assessed independently, and its effect is discussed subsequently). Given the expected correlation between RPE-coupled activation and win- or loss-coupled activation with many paradigms and analysis methods (Rohe et al., 2012; Wilson and Niv, 2015), individual differences in lambda remains a possible explanation for a wide variety of individual differences effects in reward-related activity that have been reported in the literature, as well as in response to pharmacological manipulations (Pessiglione et al., 2006).

Further evidence that these simulations are neurophysiologically plausible was obtained from analysis of the test retest reliability of the RPE and derivative-coupled beta parameters. These were in the “poor” range—levels which would generally be considered too low for psychometric work, but highly consistent with meta-analytic estimates from the literature (Elliott et al., 2020). Although the simulations included considerable noise, it is somewhat surprising that such low values were seen, as identical parameters were used to generate the data for both runs (e.g., lambda, alpha). The large effect sizes relating individual differences in lambda to RPE-coupled beta parameters that were observed (see Section 3.3, Figure 4) can be supported with such low task reliability only because the underlying relationship between these two variables was very strong, and the reliability of lambda was perfect. In practice, it is likely that there would be further reductions in these associations due to natural state-related variation in lambda for example. Some arguments can be made that the simulations might slightly underestimate ICCs of fMRI data—perhaps efficient noise reduction or higher SNR sequences might be beneficial, and it is likely that the use of random reward probabilities contributed to the low ICCs. Overall, the findings are mostly consistent with the suggestion that brain/behavior relationships should be considered medium sized effects *a priori* (Poldrack et al., 2017), but that judicious paradigm design and efficient denoising might open the potential for large effects to be observed.

There are two main contributions of the work. The first is to show the importance of lambda, rather than alpha, in determining the magnitude of the basic RPE-response. While in practice, distinctions between the two parameters have not been widely investigated in human fMRI studies, they reflect fundamentally different aspects of reinforcement learning which may be relevant for psychiatry. For example, while purchasing alcohol is legal in most countries and the majority of individuals drink in some form, a minority of individuals go on to drink excessively and develop criteria for alcohol use disorder (AUD) (Grant et al., 2015). The difference between alpha and lambda can be used to understand this variation: all adult drinkers have the capacity to form predictive associations about alcoholic beverages, and the speed of formations of these associations would be controlled by alpha. In the case of dependent drinkers, average daily consumption would reach a much higher level—one which is likely to be associated with a variety of health-related problems. Here, the level of routine alcohol consumption—asymptotic drinking—would be controlled by lambda. Similar arguments could be applied to other disorders such as obsessive-compulsive disorder (OCD: Gillan et al., 2014), anxiety disorders (Lissek et al., 2005) or major depressive disorder (MDD: Huys et al., 2013) in which learning about rewards or sources of harm are systematically altered, potentially across the lifespan.

The second contribution is to introduce the potential of a derivative for modeling the shape of the learning curve, which is quite specifically related to the learning rate, alpha. Overall, the simulations suggest the presence of powerful underlying relationships between learning rate and reinforcement efficacy parameters and RPE-related activity, so enhancing signal to noise and optimizing paradigm design in light of these simulations may be valuable for mitigating issues with the test–retest reliability of fMRI. Further experimentation is needed to determine whether the extant literature has been systematically mis-estimating a true large underlying effect size due to poor psychometric properties of fMRI design, or if positive findings have been driven mostly by analytic flexibility (Lebreton et al., 2019) and publication bias. The simulations in the present work suggest that the former scenario is at least possible.

In terms of future applications, an exciting direction might be to try to identify different neural regions whose RPE responses appear to reflect the operation of different learning rates. Currently, it is typical to identify only one learning rate per participant, per task (but see Collins et al., 2017). However, several psychological models suggest the presence of different learning systems, which have different properties. The most simple contrast is between fast and slow learning systems (e.g., Balleine and Dickinson, 1998; Ashby and Maddox, 2005; Daw et al., 2005; Pasupathy and Miller, 2005; Collins et al., 2014; Perez and Dickinson, 2020): broadly, fast learning systems are thought to mediate symbolic, detailed representations of goals, and may be dependent on working memory and other cognitive processes; slow learning systems are typically characterized as incremental and automatic, and support habitual behavior. The derivative-coupled beta parameter, in combination with the RPE-coupled beta parameter, might provide a simple metric of the learning rate of a given neural region, at least within an RL framework. This method might complement another method of estimating learning rate from neural data alone which is better established, namely the relative magnitude of anticipation- and outcome-locked neural activation (Chase et al., 2015a; Luijten et al., 2017). How these different methods correspond to one another might be a fruitful area for future study.

However, at this stage I should note that using fMRI data for parameter estimation may not be straightforward in some scenarios—in fact, the very scenarios in which I anticipate the present method will have most utility. The complication of estimation using fMRI data is that often researchers use a whole-brain analysis to define regions of interest. This initial inference step can lead to considerable bias from an estimation point of view: while voxels defined by this step may refute the null hypothesis of no difference, this inference step would select voxels with the strongest effects, potentially capitalizing on random variation. This might over-estimate the magnitude of underlying effects and have implications for replication (Cremers et al., 2017). Thus, while I show that the derivative will have most benefit in terms of improved model fitting in regions in which alpha is most divergent from the fixed alpha value used by the RPE regressor, parameter estimates from regions identified using a whole brain analysis may be over-estimated in practice, and ideally would be confirmed in a replication sample. However, once a specific region of interest has been identified independently, other methods of parameter estimation, that may be intractable at a voxelwise level (e.g., Friston et al., 2003; Turner et al., 2013), could be brought to bear.

Further relevant findings from an estimation point of view are the potential to use RPE or derivative beta maps to estimate lambda or alpha for a given subject, perhaps to corroborate findings obtained with another task—although this mapping may be difficult in some cases (Eckstein et al., 2021). A potential possibility here might be to obtain distributed predictions across the whole brain using a method such as ridge regression (e.g., Ooi et al., 2022) to reduce the impact of noise from a given voxel or region. The present findings suggest that this type of analysis may be more effect for alpha than for lambda, given that the relationship between RPE-coupled activation and lambda is obscured when the scaling of the HRF allowed to vary randomly across individuals. However, this can be recovered if the HRF scaling parameter is included in the analysis—this may be analogous to the capacity of amplitude of low frequency fluctuations (ALFF) measures to predict variation in task-related activation (Mennes et al., 2011; Di et al., 2013; Zou et al., 2013). Putting this together then, the present techniques may provide ways to estimate RL parameters (i.e., alpha, lambda) from neural data, but this may require (1) training and test samples, (2) information from distributed neural regions and (3) independent estimates of regional hemodynamic properties.

In terms of recommendations for future methodological work, the method employing derivatives may be the preferred option over the high/low difference method. This is because it is straightforward to implement which opens possibilities for generalization to other tasks, and, at least in these simulations, orthogonal to the RPE regressor (mean HRF-convolved RPE/derivative *r* ~ = − 0.01). It also distinguishes alpha-related loading on the derivative-coupled beta parameter from lambda-related loading on the main RPE-coupled beta parameter, although alpha did show some correlation with the latter. In practice, this was a modest effect size, but it does underscore the difficulty of distinguishing alpha and lambda (Chase, 2021). An important benefit of the derivative approach is that it would seem straightforwardly applicable to other types of parametric modulator: one could even imagine it being used for reaction times.

While the difference method gave the same pattern of findings overall as the derivative method, the correlation between the difference and the mean regressors was quite high, which necessitated orthogonalization. Widely-used fMRI software differ with regard to the orthogonalization of parametric regressors, and there are drawbacks regarding interpretability of this procedure when the correlations between regressors is high (Mumford et al., 2015). A second weakness of this approach is that the selection of values for the high and low learning rates was essentially arbitrary. This need not be the case for the derivative method: for example, a group or individual estimation of alpha could be performed to estimate the initial learning rate, rather than a fixed learning rate, before the derivatives are calculated.

## 5 Limitations

One underlying assumption of this work is the notion that *more* RPE neural activation is associated with a greater psychological RPE signal. While this assumption—of an absolute scale—might appear plausible, even obvious, it may not in fact hold for RPE signals: a point discussed in detail by Lebreton et al. (2019). Specifically, studies of prediction error responses in the midbrain have demonstrated a rescaling of RPE signals in midbrain dopamine neurons with local reward distributions (Tobler et al., 2005). RPEs are therefore computed relative to local reward distributions, so that the maximum neural RPE elicited scales approximately with the range of rewards available in that context. This type of finding is divergent from our assumption of an absolute scaling between psychological and neural RPE. In an individual differences context, it remains unclear how lambda might be reflected in neural activity (although see, e.g., Kirschner et al., 2016). If neural activation is perfectly normalized within individuals to a relative scale, any between-subject variation in reward activation might simply be reflective of an irrelevant dimension such as the shape or hemodynamic properties of the region. Nevertheless, it remains possible that there might be within-subject relative scaling, but between-subject absolute scaling (i.e., that more reward sensitive individuals can show a wider range of RPE-coupled BOLD signal, but that RPE signals will still adapt to contextual reward rates). It should be noted that recent evidence has suggested that intrinsic motivation may lead to an analogous set of findings to those predicted by adaptive coding models (Molinaro and Collins, 2023). The capacity for intrinsic motivation also might vary across individuals (Blain et al., 2023), leading to an alternative set of predictions generated by intrinsic motivation models, which could be pursued in future work.

Another scenario I did not consider was whether alpha influences the magnitude of the neural RPE signal directly (as opposed to indirectly by affecting prior expectations). This assumption is consistent with evidence from midbrain dopamine neurons (Fiorillo et al., 2003), in which RPE magnitude was intermediate at 50% contingencies. Overall, it would seem possible to generalize the present method to incorporate the predictions of different learning models, including those in which cue-outcome uncertainty can modulate learning rates (Mackintosh, 1975; Yu and Dayan, 2005; Le Pelley et al., 2016).

In general, for the purposes of the present work, I have tried to adopt a straightforward and widely used modeling throughout, and made only basic assumptions. One area where this is particularly evident is the simplicity of the biophysical basis of the simulated neuronal activation: a simple linear function of the model-derived RPE, which is then convolved with the HRF to simulate the BOLD signal. This approach was chosen as it aligns with typical fMRI analyses which are widely used throughout the literature (Poline and Brett, 2012). More sophisticated biophysical modeling of the BOLD signal is being developed (Daunizeau et al., 2011), and it would be intriguing to see the extent to which further biophysically plausible constraints added to the sensitivity of the method in the context of real fMRI data. Importantly however, I argue that the basic form of the RPE model should be similar regardless of such constraints, given the accurate relationship of RPE model predictions and real electrophysiological data (Schultz et al., 1997). Nevertheless, one obvious biophysical constraint not considered in the present work is the smaller dynamic range for negative deflections of dopamine firing (“dips”) than for positive deflections (Tobler et al., 2003). A valuable future direction might be to try to generalize the present modeling approach to capture such non-linearities resulting from the biophysical realization of dopaminergic neurons.

A final important point is the extent to which the findings are specific to the paradigm used for these simulations. 50% contingencies were chosen as they are expected to generate many prediction error events across a variety of learning rates (although in practice learned irrelevance effects might become significant: Le Pelley et al., 2016). While many RPE events per paradigm ensures that the GLM model fit would not generally hinge on a few critical trials, the random design might be in part responsible for the low ICCs which were observed. Intriguingly, Wilson and Niv (2015) present simulations to suggest that some designs might be more sensitive to misspecification of alpha than others. In this light, whether the derivatives method can provide additional benefit in more “alpha-sensitive” paradigms, and indeed what the design features of such paradigms are, might be a worthwhile topic for future investigation. The findings do show that the session duration and/or number of trials is an important determinant of the strength of the associations between the psychological variables (lambda/alpha) and the simulated neural responses, consistent with prior work (Nee, 2019), although substantial effect sizes are still present with low trial numbers.

## 6 Summary

While fitting predictions of reinforcement learning models to neuroimaging data has become widely adopted, the importance of accurate estimation of the learning rate parameter remains unclear. In the present work, I present a novel approach for use with GLM models, in which a derivative regressor is included with the standard RPE regressor. This regressor can capture unmodeled variation resulting from the misspecification of the learning rate parameter when modeling neural RPE-coupled signals, and clarifies the relationship of individual differences in reinforcement learning rate parameters with neural activation. This approach may provide utility for studies of reinforcement learning which are focused on individual differences, including studies of clinical populations characterized by aberrant reinforcement learning such as major depression, OCD, anxiety and substance use disorders.

## Data availability statement

The original contributions presented in the study are publicly available. This data can be found here: https://github.com/HenryWNChase/rl_derivatives.

## Author contributions

HC designed the study, performed the simulations and data analysis, and wrote the manuscript.

## References

[ref1] Akhrif A. Romanos M. Domschke K. Schmitt-Boehrer A. Neufang S. (2018). Fractal analysis of BOLD time series in a network associated with waiting impulsivity. Front. Physiol. 9:1378. doi: 10.3389/fphys.2018.01378, PMID: 30337880 PMC6180197

[ref2] Ashby F. G. Maddox W. T. (2005). Human category learning. Annu. Rev. Psychol. 56, 149–178. doi: 10.1146/annurev.psych.56.091103.070217 15709932

[ref3] Balleine B. (1992). Instrumental performance following a shift in primary motivation depends on incentive learning. J. Exp. Psychol. Anim. Behav. Process. 18, 236–250. doi: 10.1037/0097-7403.18.3.236, PMID: 1619392

[ref4] Balleine B. W. Dickinson A. (1998). Goal-directed instrumental action: contingency and incentive learning and their cortical substrates. Neuropharmacology 37, 407–419. doi: 10.1016/S0028-3908(98)00033-1, PMID: 9704982

[ref5] Blain B. Pinhorn I. Sharot T. (2023). Sensitivity to intrinsic rewards is domain general and related to mental health. Nat. Mental Health 1, 679–691. doi: 10.1038/s44220-023-00116-x PMC11041740 38665692

[ref6] Bradshaw C. M. Killeen P. R. (2012). A theory of behaviour on progressive ratio schedules, with applications in behavioural pharmacology. Psychopharmacology 222, 549–564. doi: 10.1007/s00213-012-2771-4, PMID: 22752382

[ref7] Bullmore E. Long C. Suckling J. Fadili J. Calvert G. Zelaya F.. (2001). Colored noise and computational inference in neurophysiological (fMRI) time series analysis: resampling methods in time and wavelet domains. Hum. Brain Mapp. 12, 61–78. doi: 10.1002/1097-0193(200102)12:2<61::AID-HBM1004>3.0.CO;2-W, PMID: 11169871 PMC6871881

[ref8] Calhoun V. D. Stevens M. C. Pearlson G. D. Kiehl K. A. (2004). fMRI analysis with the general linear model: removal of latency-induced amplitude bias by incorporation of hemodynamic derivative terms. NeuroImage 22, 252–257. doi: 10.1016/j.neuroimage.2003.12.029 15110015

[ref9] Cao Z. Bennett M. Orr C. Icke I. Banaschewski T. Barker G. J.. (2019). Mapping adolescent reward anticipation, receipt, and prediction error during the monetary incentive delay task. Hum. Brain Mapp. 40, 262–283. doi: 10.1002/hbm.24370, PMID: 30240509 PMC6865381

[ref10] Chase H. W. (2021). Computing the uncontrollable: insights from computational modelling of learning and choice in depression. Curr. Behav. Neurosci. Rep. 8, 28–37. doi: 10.1007/s40473-021-00228-7

[ref11] Chase H. W. Fournier J. C. Greenberg T. Almeida J. R. Stiffler R. Zevallos C. R.. (2015a). Accounting for dynamic fluctuations across time when examining fMRI test-retest reliability: analysis of a reward paradigm in the EMBARC study. PLoS One 10:e0126326. doi: 10.1371/journal.pone.0126326, PMID: 25961712 PMC4427400

[ref12] Chase H. W. Kumar P. Eickhoff S. B. Dombrovski A. Y. (2015b). Reinforcement learning models and their neural correlates: an activation likelihood estimation meta-analysis. Cogn. Affect. Behav. Neurosci. 15, 435–459. doi: 10.3758/s13415-015-0338-7, PMID: 25665667 PMC4437864

[ref13] Chase H. W. Loriemi P. Wensing T. Eickhoff S. B. Nickl-Jockschat T. (2018). Meta-analytic evidence for altered mesolimbic responses to reward in schizophrenia. Hum. Brain Mapp. 39, 2917–2928. doi: 10.1002/hbm.24049, PMID: 29573046 PMC6866586

[ref14] Chen G. Saad Z. S. Britton J. C. Pine D. S. Cox R. W. (2013). Linear mixed-effects modeling approach to FMRI group analysis. NeuroImage 73, 176–190. doi: 10.1016/j.neuroimage.2013.01.047, PMID: 23376789 PMC3638840

[ref15] Cohen M. X. (2007). Individual differences and the neural representations of reward expectation and reward prediction error. Soc. Cogn. Affect. Neurosci. 2, 20–30. doi: 10.1093/scan/nsl021, PMID: 17710118 PMC1945222

[ref16] Cohen J. D. Daw N. Engelhardt B. Hasson U. Li K. Niv Y.. (2017). Computational approaches to fMRI analysis. Nat. Neurosci. 20, 304–313. doi: 10.1038/nn.4499, PMID: 28230848 PMC5457304

[ref17] Collins A. G. Brown J. K. Gold J. M. Waltz J. A. Frank M. J. (2014). Working memory contributions to reinforcement learning impairments in schizophrenia. J. Neurosci. 34, 13747–13756. doi: 10.1523/JNEUROSCI.0989-14.2014, PMID: 25297101 PMC4188972

[ref18] Collins A. G. E. Ciullo B. Frank M. J. Badre D. (2017). Working memory load strengthens reward prediction errors. J. Neurosci. 37, 4332–4342. doi: 10.1523/JNEUROSCI.2700-16.2017, PMID: 28320846 PMC5413179

[ref19] Corbin N. Todd N. Friston K. J. Callaghan M. F. (2018). Accurate modeling of temporal correlations in rapidly sampled fMRI time series. Hum. Brain Mapp. 39, 3884–3897. doi: 10.1002/hbm.24218, PMID: 29885101 PMC6175228

[ref20] Cremers H. R. Wager T. D. Yarkoni T. (2017). The relation between statistical power and inference in fMRI. PLoS One 12:e0184923. doi: 10.1371/journal.pone.0184923, PMID: 29155843 PMC5695788

[ref21] Culbreth A. J. Westbrook A. Xu Z. Barch D. M. Waltz J. A. (2016). Intact ventral striatal prediction error signaling in medicated schizophrenia patients. Biol. Psychiatry Cogn. Neurosci. Neuroimaging 1, 474–483. doi: 10.1016/j.bpsc.2016.07.007, PMID: 28239676 PMC5321567

[ref22] Daunizeau J. David O. Stephan K. E. (2011). Dynamic causal modelling: a critical review of the biophysical and statistical foundations. NeuroImage 58, 312–322. doi: 10.1016/j.neuroimage.2009.11.062 19961941

[ref23] Davey C. E. Grayden D. B. Egan G. F. Johnston L. A. (2013). Filtering induces correlation in fMRI resting state data. NeuroImage 64, 728–740. doi: 10.1016/j.neuroimage.2012.08.022, PMID: 22939874

[ref24] Daw N. D. (2009). “Trial-by-trial data analysis using computational models” in Decision making, affect and learning. eds. Delgado M. R. Phelps E. A. Robbins T. W. (Oxford, UK: Oxford University Press), 3–38.

[ref25] Daw N. D. Niv Y. Dayan P. (2005). Uncertainty-based competition between prefrontal and dorsolateral striatal systems for behavioral control. Nat. Neurosci. 8, 1704–1711. doi: 10.1038/nn1560, PMID: 16286932

[ref26] Di X. Kannurpatti S. S. Rypma B. Biswal B. B. (2013). Calibrating BOLD fMRI activations with neurovascular and anatomical constraints. Cereb. Cortex 23, 255–263. doi: 10.1093/cercor/bhs001, PMID: 22345358 PMC3539449

[ref27] Diedenhofen B. Musch J. (2015). Cocor: a comprehensive solution for the statistical comparison of correlations. PLoS One 10:e0121945. doi: 10.1371/journal.pone.0121945, PMID: 25835001 PMC4383486

[ref28] Dunn O. J. Clark V. A. (1971). Comparison of tests of the equality of dependent correlation. J. Am. Stat. Assoc. 66, 904–908. doi: 10.1080/01621459.1971.10482369

[ref29] Eckstein M. K. Wilbrecht L. Collins A. G. E. (2021). What do reinforcement learning models measure? Interpreting model parameters in cognition and neuroscience. Curr. Opin. Behav. Sci. 41, 128–137. doi: 10.1016/j.cobeha.2021.06.004, PMID: 34984213 PMC8722372

[ref30] Elliott M. L. Knodt A. R. Ireland D. Morris M. L. Poulton R. Ramrakha S.. (2020). What is the test-retest reliability of common task-functional MRI measures? New empirical evidence and a Meta-analysis. Psychol. Sci. 31, 792–806. doi: 10.1177/0956797620916786, PMID: 32489141 PMC7370246

[ref31] Fiorillo C. D. Tobler P. N. Schultz W. (2003). Discrete coding of reward probability and uncertainty by dopamine neurons. Science 299, 1898–1902. doi: 10.1126/science.1077349, PMID: 12649484

[ref32] Fouragnan E. Retzler C. Philiastides M. G. (2018). Separate neural representations of prediction error valence and surprise: evidence from an fMRI meta-analysis. Hum. Brain Mapp. 39, 2887–2906. doi: 10.1002/hbm.24047, PMID: 29575249 PMC6866507

[ref33] Fournier J. C. Chase H. W. Almeida J. Phillips M. L. (2014). Model specification and the reliability of fMRI results: implications for longitudinal neuroimaging studies in psychiatry. PLoS One 9:e105169. doi: 10.1371/journal.pone.0105169, PMID: 25166022 PMC4148299

[ref34] Frank M. J. Moustafa A. A. Haughey H. M. Curran T. Hutchison K. E. (2007). Genetic triple dissociation reveals multiple roles for dopamine in reinforcement learning. Proc. Natl. Acad. Sci. U. S. A. 104, 16311–16316. doi: 10.1073/pnas.0706111104 17913879 PMC2042203

[ref35] Frassle S. Lomakina E. I. Razi A. Friston K. J. Buhmann J. M. Stephan K. E. (2017). Regression DCM for fMRI. NeuroImage 155, 406–421. doi: 10.1016/j.neuroimage.2017.02.090 28259780

[ref36] Friston K. J. Fletcher P. Josephs O. Holmes A. Rugg M. D. Turner R. (1998). Event-related fMRI: characterizing differential responses. NeuroImage 7, 30–40. doi: 10.1006/nimg.1997.0306 9500830

[ref37] Friston K. J. Harrison L. Penny W. (2003). Dynamic causal modelling. NeuroImage 19, 1273–1302. doi: 10.1016/S1053-8119(03)00202-7 12948688

[ref38] Garrison J. Erdeniz B. Done J. (2013). Prediction error in reinforcement learning: a meta-analysis of neuroimaging studies. Neurosci. Biobehav. Rev. 37, 1297–1310. doi: 10.1016/j.neubiorev.2013.03.023, PMID: 23567522

[ref39] Gillan C. M. Morein-Zamir S. Urcelay G. P. Sule A. Voon V. Apergis-Schoute A. M.. (2014). Enhanced avoidance habits in obsessive-compulsive disorder. Biol. Psychiatry 75, 631–638. doi: 10.1016/j.biopsych.2013.02.002, PMID: 23510580 PMC3988923

[ref40] Grady C. L. Rieck J. R. Nichol D. Rodrigue K. M. Kennedy K. M. (2021). Influence of sample size and analytic approach on stability and interpretation of brain-behavior correlations in task-related fMRI data. Hum. Brain Mapp. 42, 204–219. doi: 10.1002/hbm.25217, PMID: 32996635 PMC7721240

[ref41] Granger K. T. Ferrar J. Caswell S. Haselgrove M. Moran P. M. Attwood A.. (2021). Effects of 7.5% carbon dioxide and nicotine administration on latent inhibition. Front. Psych. 12:582745. doi: 10.3389/fpsyt.2021.582745 PMC8085318 33935819

[ref42] Grant B. F. Goldstein R. B. Saha T. D. Chou S. P. Jung J. Zhang H.. (2015). Epidemiology of DSM-5 alcohol use disorder: results from the National Epidemiologic Survey on alcohol and related conditions III. JAMA Psychiatry 72, 757–766. doi: 10.1001/jamapsychiatry.2015.0584, PMID: 26039070 PMC5240584

[ref43] Handwerker D. A. Ollinger J. M. D'esposito M. (2004). Variation of BOLD hemodynamic responses across subjects and brain regions and their effects on statistical analyses. NeuroImage 21, 1639–1651. doi: 10.1016/j.neuroimage.2003.11.029, PMID: 15050587

[ref44] Hogarth L. Chase H. W. (2012). Evaluating psychological markers for human nicotine dependence: tobacco choice, extinction, and Pavlovian-to-instrumental transfer. Exp. Clin. Psychopharmacol. 20, 213–224. doi: 10.1037/a0027203 22369668

[ref45] Hursh S. R. Silberberg A. (2008). Economic demand and essential value. Psychol. Rev. 115, 186–198. doi: 10.1037/0033-295X.115.1.186 18211190

[ref46] Huys Q. J. Pizzagalli D. A. Bogdan R. Dayan P. (2013). Mapping anhedonia onto reinforcement learning: a behavioural meta-analysis. Biol. Mood Anxiety Disord. 3:12. doi: 10.1186/2045-5380-3-12, PMID: 23782813 PMC3701611

[ref47] Janouschek H. Chase H. W. Sharkey R. J. Peterson Z. J. Camilleri J. A. Abel T.. (2021). The functional neural architecture of dysfunctional reward processing in autism. Neuroimage Clin. 31:102700. doi: 10.1016/j.nicl.2021.102700 34161918 PMC8239466

[ref48] Katahira K. Toyama A. (2021). Revisiting the importance of model fitting for model-based fMRI: it does matter in computational psychiatry. PLoS Comput. Biol. 17:e1008738. doi: 10.1371/journal.pcbi.1008738, PMID: 33561125 PMC7899379

[ref49] Keren H. O’Callaghan G. Vidal-Ribas P. Buzzell G. A. Brotman M. A. Leibenluft E.. (2018). Reward processing in depression: a conceptual and Meta-analytic review across fMRI and EEG studies. Am. J. Psychiatry 175, 1111–1120. doi: 10.1176/appi.ajp.2018.17101124, PMID: 29921146 PMC6345602

[ref50] Kirschner M. Hager O. M. Bischof M. Hartmann-Riemer M. N. Kluge A. Seifritz E.. (2016). Deficits in context-dependent adaptive coding of reward in schizophrenia. NPJ Schizophr. 2:16020. doi: 10.1038/npjschz.2016.20, PMID: 27430009 PMC4945098

[ref51] Kumar P. Waiter G. Ahearn T. Milders M. Reid I. Steele J. D. (2008). Abnormal temporal difference reward-learning signals in major depression. Brain 131, 2084–2093. doi: 10.1093/brain/awn136, PMID: 18579575

[ref52] Lawson R. P. Nord C. L. Seymour B. Thomas D. L. Dayan P. Pilling S.. (2017). Disrupted habenula function in major depression. Mol. Psychiatry 22, 202–208. doi: 10.1038/mp.2016.81, PMID: 27240528 PMC5285459

[ref53] Le Pelley M. E. Mitchell C. J. Beesley T. George D. N. Wills A. J. (2016). Attention and associative learning in humans: an integrative review. Psychol. Bull. 142, 1111–1140. doi: 10.1037/bul0000064, PMID: 27504933

[ref54] Lebreton M. Bavard S. Daunizeau J. Palminteri S. (2019). Assessing inter-individual differences with task-related functional neuroimaging. Nat. Hum. Behav. 3, 897–905. doi: 10.1038/s41562-019-0681-8, PMID: 31451737

[ref55] Lissek S. Powers A. S. Mcclure E. B. Phelps E. A. Woldehawariat G. Grillon C.. (2005). Classical fear conditioning in the anxiety disorders: a meta-analysis. Behav. Res. Ther. 43, 1391–1424. doi: 10.1016/j.brat.2004.10.007, PMID: 15885654

[ref56] Luijten M. Schellekens A. F. Kuhn S. Machielse M. W. Sescousse G. (2017). Disruption of reward processing in addiction: an image-based Meta-analysis of functional magnetic resonance imaging studies. JAMA Psychiatry 74, 387–398. doi: 10.1001/jamapsychiatry.2016.3084 28146248

[ref57] Mackintosh N. J. (1975). A theory of attention: variations in the associability of stimuli with reinforcement. Psychol. Rev. 82, 276–298. doi: 10.1037/h0076778

[ref58] Madsen H. B. Ahmed S. H. (2015). Drug versus sweet reward: greater attraction to and preference for sweet versus drug cues. Addict. Biol. 20, 433–444. doi: 10.1111/adb.12134 24602027

[ref59] Mennes M. Zuo X. N. Kelly C. Di Martino A. Zang Y. F. Biswal B.. (2011). Linking inter-individual differences in neural activation and behavior to intrinsic brain dynamics. NeuroImage 54, 2950–2959. doi: 10.1016/j.neuroimage.2010.10.046, PMID: 20974260 PMC3091620

[ref60] Molinaro G. Collins A. G. E. (2023). Intrinsic rewards explain context-sensitive valuation in reinforcement learning. PLoS Biol. 21:e3002201. doi: 10.1371/journal.pbio.3002201 37459394 PMC10374061

[ref61] Monti M. M. (2011). Statistical analysis of fMRI time-series: a critical review of the GLM approach. Front. Hum. Neurosci. 5:28. doi: 10.3389/fnhum.2011.00028 21442013 PMC3062970

[ref62] Mumford J. A. Poline J. B. Poldrack R. A. (2015). Orthogonalization of regressors in FMRI models. PLoS One 10:e0126255. doi: 10.1371/journal.pone.0126255, PMID: 25919488 PMC4412813

[ref63] Murray G. K. Corlett P. R. Clark L. Pessiglione M. Blackwell A. D. Honey G.. (2008). Substantia nigra/ventral tegmental reward prediction error disruption in psychosis. Mol. Psychiatry 13, 267–276. doi: 10.1038/sj.mp.4002058, PMID: 17684497 PMC2564111

[ref64] Nee D. E. (2019). fMRI replicability depends upon sufficient individual-level data. Commun. Biol. 2:130. doi: 10.1038/s42003-019-0378-6, PMID: 30993214 PMC6461660

[ref65] Neumann D. L. Waters A. M. (2006). The use of an unpleasant sound as an unconditional stimulus in a human aversive Pavlovian conditioning procedure. Biol. Psychol. 73, 175–185. doi: 10.1016/j.biopsycho.2006.03.004 16698165

[ref66] O'Doherty J. P. Dayan P. Friston K. Critchley H. Dolan R. J. (2003). Temporal difference models and reward-related learning in the human brain. Neuron 38, 329–337. doi: 10.1016/S0896-6273(03)00169-7 12718865

[ref67] O'Doherty J. P. Hampton A. Kim H. (2007). Model-based fMRI and its application to reward learning and decision making. Ann. N. Y. Acad. Sci. 1104, 35–53. doi: 10.1196/annals.1390.022, PMID: 17416921

[ref68] Olszowy W. Aston J. Rua C. Williams G. B. (2019). Accurate autocorrelation modeling substantially improves fMRI reliability. Nat. Commun. 10:1220. doi: 10.1038/s41467-019-09230-w, PMID: 30899012 PMC6428826

[ref69] Ooi L. Q. R. Chen J. Shaoshi Z. Kong R. Tam A. Li J.. (2022). Comparison of individualized behavioral predictions across anatomical, diffusion and functional connectivity MRI. NeuroImage 263:119636. doi: 10.1016/j.neuroimage.2022.119636 36116616

[ref70] Pasupathy A. Miller E. K. (2005). Different time courses of learning-related activity in the prefrontal cortex and striatum. Nature 433, 873–876. doi: 10.1038/nature03287, PMID: 15729344

[ref71] Perez O. D. Dickinson A. (2020). A theory of actions and habits: the interaction of rate correlation and contiguity systems in free-operant behavior. Psychol. Rev. 127, 945–971. doi: 10.1037/rev0000201, PMID: 32406713

[ref72] Pessiglione M. Seymour B. Flandin G. Dolan R. J. Frith C. D. (2006). Dopamine-dependent prediction errors underpin reward-seeking behaviour in humans. Nature 442, 1042–1045. doi: 10.1038/nature05051, PMID: 16929307 PMC2636869

[ref73] Poldrack R. A. Baker C. I. Durnez J. Gorgolewski K. J. Matthews P. M. Munafo M. R.. (2017). Scanning the horizon: towards transparent and reproducible neuroimaging research. Nat. Rev. Neurosci. 18, 115–126. doi: 10.1038/nrn.2016.167, PMID: 28053326 PMC6910649

[ref74] Poline J. B. Brett M. (2012). The general linear model and fMRI: does love last forever? NeuroImage 62, 871–880. doi: 10.1016/j.neuroimage.2012.01.133, PMID: 22343127

[ref75] Radua J. Schmidt A. Borgwardt S. Heinz A. Schlagenhauf F. Mcguire P.. (2015). Ventral striatal activation during reward processing in psychosis: a Neurofunctional Meta-analysis. JAMA Psychiatry 72, 1243–1251. doi: 10.1001/jamapsychiatry.2015.2196, PMID: 26558708

[ref76] Rescorla R. A. Wagner A. R. (1972). “A theory of Pavlovian conditioning: variations in the effectiveness of reinforcement and nonrerinforcement” in Classical conditioning II: Current research and theory. ed. Prokasy A. H. B. W. F. (New York: Appleton-Century-Crofts), 64–99.

[ref77] Rodgers R. J. Holch P. Tallett A. J. (2010). Behavioural satiety sequence (BSS): separating wheat from chaff in the behavioural pharmacology of appetite. Pharmacol. Biochem. Behav. 97, 3–14. doi: 10.1016/j.pbb.2010.03.001, PMID: 20214921

[ref78] Rohe T. Weber B. Fliessbach K. (2012). Dissociation of BOLD responses to reward prediction errors and reward receipt by a model comparison. Eur. J. Neurosci. 36, 2376–2382. doi: 10.1111/j.1460-9568.2012.08125.x, PMID: 22595033

[ref79] Rose E. J. Salmeron B. J. Ross T. J. Waltz J. Schweitzer J. B. McClure S. M.. (2014). Temporal difference error prediction signal dysregulation in cocaine dependence. Neuropsychopharmacology 39, 1732–1742. doi: 10.1038/npp.2014.21, PMID: 24569319 PMC4023147

[ref80] Schonberg T. O’Doherty J. P. Joel D. Inzelberg R. Segev Y. Daw N. D. (2010). Selective impairment of prediction error signaling in human dorsolateral but not ventral striatum in Parkinson's disease patients: evidence from a model-based fMRI study. NeuroImage 49, 772–781. doi: 10.1016/j.neuroimage.2009.08.011 19682583

[ref81] Schultz W. Dayan P. Montague P. R. (1997). A neural substrate of prediction and reward. Science 275, 1593–1599. doi: 10.1126/science.275.5306.1593 9054347

[ref82] Stoops W. W. Lile J. A. Fillmore M. T. Glaser P. E. Rush C. R. (2005). Reinforcing effects of modafinil: influence of dose and behavioral demands following drug administration. Psychopharmacology 182, 186–193. doi: 10.1007/s00213-005-0044-1, PMID: 15986191

[ref83] Sutton R. S. Barto A. G. (2018). Reinforcement learning: An introduction. Cambridge, MA, USA: MIT press.

[ref84] Tobler P. N. Dickinson A. Schultz W. (2003). Coding of predicted reward omission by dopamine neurons in a conditioned inhibition paradigm. J. Neurosci. 23, 10402–10410. doi: 10.1523/JNEUROSCI.23-32-10402.2003, PMID: 14614099 PMC6741002

[ref85] Tobler P. N. Fiorillo C. D. Schultz W. (2005). Adaptive coding of reward value by dopamine neurons. Science 307, 1642–1645. doi: 10.1126/science.1105370 15761155

[ref86] Turner B. M. Forstmann B. U. Wagenmakers E. J. Brown S. D. Sederberg P. B. Steyvers M. (2013). A Bayesian framework for simultaneously modeling neural and behavioral data. NeuroImage 72, 193–206. doi: 10.1016/j.neuroimage.2013.01.048, PMID: 23370060 PMC4140412

[ref87] Wilson R. C. Niv Y. (2015). Is model fitting necessary for model-based fMRI? PLoS Comput. Biol. 11:e1004237. doi: 10.1371/journal.pcbi.1004237 26086934 PMC4472514

[ref88] Yu A. J. Dayan P. (2005). Uncertainty, neuromodulation, and attention. Neuron 46, 681–692. doi: 10.1016/j.neuron.2005.04.026 15944135

[ref89] Zou Q. Ross T. J. Gu H. Geng X. Zuo X. N. Hong L. E.. (2013). Intrinsic resting-state activity predicts working memory brain activation and behavioral performance. Hum. Brain Mapp. 34, 3204–3215. doi: 10.1002/hbm.22136, PMID: 22711376 PMC6870161

