# Supplementary material for: A novel technique for delineating the effect of variation in the learning rate on the neural correlates of reward prediction errors in model-based fMRI
Source: Front Psychol. 2023 Dec 21;14:1211528. doi: 10.3389/fpsyg.2023.1211528 (PMC10768009; doi:10.3389/fpsyg.2023.1211528)
Supplement: Supplementary file 1 [file Table_1.docx]

|  | Lambda / RPE  Association | Alpha / RPE  Association | Lambda / Derivative  Association | Alpha / Derivative  Association |
| --- | --- | --- | --- | --- |
| Pavlovian / Fixed LR (0.2) / No derivative | T=201.39 | T=-45.53 | N/A | N/A |
| Instrumental / Fixed LR (0.2) / No derivative | T=173.52 | T=-31.041 | N/A | N/A |
| Pavlovian / Individualized LR, high precision / No derivative | T=177.066 | T=27.27 | N/A | N/A |
| Pavlovian / Individualized LR, low precision / No derivative | T=181.023 | T=31.45 | N/A | N/A |
| Pavlovian / Fixed LR / Gradient derivative | T=120.88 | T=34.99 | T<1.15 | T=395.44 |
| Pavlovian / Fixed LR / Gradient derivative / win, no win regressor | T=155.66 | T=7.34 | T=87.040 | T=343.66 |
| Instrumental / Fixed LR / Gradient derivative | T=152.29 | T=30.18 | T=2.55 | T=275.69 |
| Pavlovian / Fixed LR / Difference derivative | T=183.28 | T=20.98 | T=6.80 | T=395.88 |

Supplementary Table 1: Summary of analyses run in sections 3.1 and 3.2
